# Qualitative (pure) mathematics as an alternative to measurement

**DOI:** 10.3389/fpsyg.2024.1374308

**Published:** 2024-10-02

**Authors:** Václav Linkov

**Affiliations:** Institute of Applied Psychology, Faculty of Social and Economic Sciences, Comenius University in Bratislava, Bratislava, Slovakia

**Keywords:** measurement, qualitative mathematics, quantification, psychology, non-numerical, mathematics education

## Abstract

This paper focuses on the possible usage of qualitative mathematics in psychology. Qualitative mathematics is understood to be equivalent to pure mathematics. First, it is explained that mathematics is a discipline studying patterns in reproducible mental objects. Qualitative mathematics is presented as an alternative to measurement, potentially offering the same level of exactness, clarity, and rigor. This perspective might lead psychologists to explore connections between a phenomenon and any kind of mathematical structure, regardless of whether the structure is quantitative. Usage of (any) mathematical structures might require scholars who are familiar with them. Consequently, changes in mathematics education may also be needed. Introducing non-numerical structures into mathematics education—thereby partially revisiting the New Math Movement—could train individuals more prepared for a creative approach to the use of structures and less inclined to view everything as quantitative.

## 1 Introduction

There is a long-term debate in psychology about whether all or nearly all psychological phenomena should be quantified and studied using quantitative methods, or if quantification is not a suitable method for the majority of psychological attributes (Toomela, 2008; Uher, 2021; Franz, 2022). Quantification is a topic for psychologists, as quantitative structure is the only part of mathematics typically used in psychology. In this paper I first attempt to explain what qualitative mathematics is. Second, I argue that if qualitative mathematics is to be used, it could serve as an alternative to quantification and measurement, offering the same level of exactness. Third, I argue that this would need change in mathematics education, which primarily focuses on numerical representations in schools.

## 2 Qualitative mathematics

Lee (2013) introduced the term “qualitative mathematics” in the title of his book but did not define it there. This term is not frequently used by mathematicians, and there is no universally accepted definition in mathematics either. However, a commonly accepted meaning among many mathematicians might be that “qualitative mathematics” is synonymous with “pure mathematics.” I will elaborate on what this means and the consequences of this interpretation of “qualitative mathematics.”

Defining (pure) mathematics is not straightforward. Byers (2017) believes that the best description of mathematics is that it is what mathematicians do. In a modern view, we might define mathematics as the science of patterns (Devlin, 2012). Another definition might be that offered by Hersh (2014), who states that mathematics involves ideas, concepts, which exists only in the shared consciousness of human beings… is both a science and a “humanity” “(Hersh, 2014, p. 163). He describes it as a discipline studying “mental objects with reproducible properties” (p. 163).

An important characteristic of mathematics is its frequent need to clarify and change terminology until it finds some representation of a problem that allows it to be solved. Ziegler and Loos (2014, p. 1210) state that people are usually not aware that part of mathematics “is a struggle to find and shape the ‘right’ concepts/definitions and to pose/develop the ‘right’ questions and problems.” This notion is further developed by Schwartz (2006, p. 232): “Mathematics must deal with well-defined situations. Thus, in its relations with science, mathematics depends on an intellectual effort outside of mathematics for the crucial specification of the approximation which mathematics is to take literally.” This “well-defined” does not mean that terminology must be precisely defined. When Newton and Leibniz developed calculus, they did not have precisely defined terminology for “continuous,” and they relied on intuitive understanding—this term was precisely defined by Bolzano more than 100 years later (Boyer, 1949). Formalizing a problem into precise terminology can be difficult, and some mathematicians believe that the most important part of problem-solving involves unconscious processes (Hadamard, 1945).

Let us consider some relations between mathematics and psychology. William James defines psychological phenomena as “such things as we call feelings, desires, cognitions, reasonings, decisions, and the like” (James, 1950, p. 1). Here we see that both mathematics and psychology study objects that exist only in the human mind. Since Cantor (1895, p. 481) defined a set as “any collection M of definite, well-distinguished objects m of our perception or our thought,” psychological phenomena might also be considered as a set (if they are “well-distinguished”). A logical consequence might be to look for similarities between mathematical and psychological objects so that mathematical objects could be used as representations of psychological ones.

If we interpret “qualitative mathematics” to mean “pure mathematics,” the counterpart to this is applied mathematics. Both disciplines deal with mathematical objects as their subject matter, but their objectives and approaches differ. Higham (2015, p. 1), in attempting to describe what this difference in objectives and approaches entails, notes that defining it is nearly impossible; hence, he cites the perspectives of several scholars without providing a concrete source. Applied mathematics could be described as “the bridge connecting pure mathematics with science and technology,” according to William Prager. Richard Courant offers a deeper insight, stating that “Applied mathematics is not a definable scientific field but a human attitude… [the scientist] must be willing to make compromises regarding rigorous mathematical completeness.” The third perspective that Higham includes is from Peter Lax, who remarks that “the applied mathematician must rely on… special solutions, asymptotic descriptions, simplified equations, experimentation both in the laboratory and on the computer.” The main difference in objectives is that while pure mathematics focuses on theoretical understanding, applied mathematics is concerned with practical applications in the external world. The difference in approach is that pure mathematics seeks to comprehend why something is valid, whereas applied mathematics is satisfied if it provides reproducible results. Applied mathematics does not concern itself with understanding the underlying reasons, thus it is less reflective of the theoretical aspects.

Understanding that “qualitative mathematics” encompasses all mathematical objects is evident in Lee’s (2013) book. One chapter discusses complex dynamical systems, which are systems utilizing nonlinear functions over a quantitative structure. These systems necessitate the measurement of quantitative variables. In Lee’s text, the quantitative aspect is merely one instance of the qualitative. What characterizes mathematics as qualitative is the perspective it adopts. The crucial factor is whether the mathematical structure aligns with a psychological phenomenon. A useful term describing the opposite of this attitude is “*opportunist mathematics*.” Stöltzner (2004) asserts that when a scientific discipline has only a weak theory of itself and poorly defined terminology, applied mathematicians adopt a strategy of *mathematical opportunism* towards this discipline. This means they engineer situations where they can apply their preferred mathematical structures to represent some phenomenon from the discipline, disregarding the phenomenon’s internal structure to facilitate this engineering. Psychology, being a discipline with a weak theoretical foundation, has witnessed such engineering attempts by mathematical opportunists especially when it comes to statistics—an example is Charles Sperman who did not verify whether the attributes he considered quantitative truly possessed a quantitative structure (Michell, 2023). However, opportunism is not a characteristic exclusive to statistics. The mathematical structures presented in Lee’s (2013) book may be utilized with the same degree of opportunism. In relation to psychology, opportunistic mathematics can be defined as mathematics that does not respect the structure of psychological phenomena.

Let us summarize this section: qualitative, or pure, mathematics is a discipline that seeks patterns in reproducible mental objects, sometimes employing imprecise terminology with the hope of refining it in the future. It differs from its counterpart, applied mathematics, in that it does not make compromises regarding the mutual relations of the mental objects it studies, which should be consistent with each other.

## 3 Qualitative mathematics as an alternative to measurement

Quantitative measurement attracts scholars due to its exactness, precision, rigor, and clarity (Michell, 1999:34; Gould, 1996). It also enables the standardization of processes and objective decision-making for governments (Porter, 1995). However, it has also faced sharp criticism from many scholars in psychology. Some psychologists think that quantitative models might not describe the psychological phenomena well (Guyon et al., 2018). Psychologists therefore complain that numerical measurement suitable for physics is not suitable for psychology (Trendler, 2009; Slaney, 2023), and question the application of the same rules used in physical sciences to psychology (Tafreshi, 2022). Some scholars think that regarding its mathematization, psychology should broaden its scope beyond just quantitative approaches (Omi, 2012). Michell (2003) suggests that quantitative attributes should not be the sole focus of scientific inquiry, advocating for the exploration of non-quantitative structures when evidence for quantitativeness is lacking. If no quantitative structure is found, it should be seen as “the beginning of the search for the kind of non-quantitative structure in which nature, in this instance, is arranged” (Michell, 2003:531). Barrett (2003) suggests that graphs, language grammar or automata might be employed as non-quantitative structures when doing structural analysis of data. Some critics of measurement might view qualitative mathematics as a potential alternative, offering the same level of exactness, rigor, and clarity. I will elaborate on this possibility in the following paragraphs.

The use of qualitative mathematics, as described in Lee’s (2013) book, likely requires the adoption of some form of structuralism, which posits the existence of inherent mathematical structures within the objects of psychological phenomena. In my opinion, assigning a member of a quantitative set during the (quantitative) measurement process is a similar activity to assigning a member of any other set in qualitative mathematics. The difference lies only in the type of properties that need to be evaluated. The use of qualitative mathematics would therefore require assigning elements of a structure (a set with specific properties) to certain attributes of the perceived phenomenon and evaluating whether these attributes satisfy those properties. Assigning a member of a mathematical set to some aspect of the measured phenomenon would require a human interpreter trained to conduct this measurement (Millikan, 2021). The interpreter must maintain contact with the actual phenomenon to avoid reifying the mathematical representation and using operations that are available in this representation but not applicable to the real phenomenon (Uher, 2023; Linkov, 2021).

According to metrologists, measurement needs to define the objects under measurement, the property to be measured, and the measurands. There should also be reproducibility in the measurement process—the same conditions should always produce the same measurement result. The measurement should be subject-independent, meaning the same conditions should yield the same result regardless of who is measuring (Uher, 2020). Measurement should also adhere to data generation traceability, so it should be traceable how the measurement result was produced in a specific case (Uher, 2022). In my opinion, all these requirements can be met for any mathematical structure because reproducibility, the most crucial of these requirements, is a necessary condition for something to be mathematizable. Therefore, qualitative mathematics structures might offer the same level of clarity as measurement and could serve as its alternative.

It should be noted that the term “qualitative” has different meanings in “qualitative mathematics” and “qualitative measurement” as used in metrology (Pendrill and Petersson, 2016). In metrology, “qualitative measurement” refers to simpler structures, such as nominal or ordinal scales, whereas in “qualitative mathematics,” it encompasses any structure, which can be highly complex. It is also important to clarify what constitutes the similarity between “qualitative” in “qualitative research” and in “qualitative mathematics.” Aspers and Corte (2019, p. 155) define qualitative research as “an iterative process in which improved understanding for the scientific community is achieved by making new significant distinctions resulting from getting closer to the phenomenon studied.” In other words, it involves spending time speculating about the object being studied to uncover its specific characteristics. This is similar to mathematics, because mathematics is often considered a struggle to find the right concepts and definitions (Ziegler and Loos, 2014). The similarity between “qualitative” in “qualitative research” and in “qualitative mathematics” lies in the way researchers think, not in the structures being investigated.

While laypeople might assume that mathematics is a discipline of clear concepts and definite algorithms, a more accurate description would be a discipline that seeks to resolve ambiguities arising from incompatible frames of reference of certain concepts (which may themselves be clear). This resolution process can take hundreds of years (Byers, 2007, p. 28). The structures produced by mathematicians and the distinctions made by qualitative researchers represent two such frames of reference. Quantitative research draws much of its strength from the rigor and clarity of quantitative structures. However, if qualitative researchers hope to apply “qualitative” mathematical structures in the same straightforward manner, there is no easy solution. Establishing a correspondence between a mathematical structure and the phenomenon being studied requires a deeper understanding of both the phenomenon and the structure. Qualitative research deepens understanding of the phenomenon through the research process (Aspers and Corte, 2019), while gaining a deeper grasp of mathematical structures may require education in these structures.

## 4 Mathematical intuition might need changes in education

A crucial question concerning the use of qualitative mathematics in the social sciences is how to determine whether there is a mathematical structure that can effectively represent a social science phenomenon. This process is akin to searching for a morphism between the mathematical structure and the internal structure of the phenomenon, which would formalize the phenomenon. It is unlikely that any algorithm exists for conducting such a formalization. Insights from practicing mathematicians suggest that finding such a connection between two structures requires intuition. A scientist often spends time studying the phenomenon until inspiration strikes suddenly and unexpectedly (Hadamard, 1945; Fitzgerald and James, 2007). Creating mathematical knowledge involves “guessing a web of ideas, and then progressively strengthening and modifying the web until it is logically unassailable” (Ruelle, 2007, p. 114). To make educated guesses about the connections between mathematical and psychological structures using this intuition, a social scientist needs experience with qualitative mathematics, which is often lacking. High school students are predominantly taught quantitative mathematical disciplines, leading them to equate mathematization and formalization with quantification. Current high school curricula, such as those described by Jeřábek et al. (2021), are designed for technical fields and natural sciences, where quantification is suitable. However, the non-numerical qualitative mathematics that could be relevant for the human sciences is notably absent.

The use of qualitative mathematics in psychology might be facilitated if mathematics were taught as a search for rules valid within certain sets or as a study of relations between two sets. Examples of such subject matter could include teaching abstract algebra and conducting proofs to determine whether a set has the properties of a certain structure, such as a semigroup (a set with an associative binary operation), or examining morphisms between these sets. If a significant portion of high school curricula were composed of such mathematical content, graduates would be less inclined to uncritically accept the quantification of real-world phenomena and would be more inclined to explore non-numerical formalizations.

The concept of teaching mathematics as an understanding of structures was promoted by the New Math movement (NMM), whose proponents believed that “math textbooks’ and teachers’ traditional reliance on memorization and regurgitation gave students a misleading sense of what mathematicians *do* and what mathematics was *about*” (Phillips, 2015:13). Consequently, they aimed to shift the school mathematics curriculum from learning skills and facts to acquiring conceptual understanding. The NMM, based on the ideas of the French Bourbaki group of mathematicians (Munson, 2010), initially found success in the 1960s in the US, France, and many European countries (De Bock, 2023; Gosztonyi, 2015; Prytz, 2020), but ultimately its reforms were unsuccessful. The NMM sought to provide people with a solid foundation in mathematics, enabling them to apply it in various jobs (Phillips, 2015:3). Perhaps the desire to be solid was the reason why the new math movement was unsuccessful, as parents resisted the changes, preferring that schools continue to focus on drilling students (p. 19).

NMM failed because its curriculum did not effectively train individuals in computation (Phillips, 2015:5), but psychology does not require such a drastic curriculum change as the cessation of computation drills. What psychology and social sciences might need is not necessarily solidity in the mathematical sense and teaching a deep understanding of structures, but rather instructing individuals to recognize the many possible sets that could serve as the mathematization of something.

## 5 Discussion

I have previously mentioned that what qualifies mathematics as qualitative, especially when used to represent psychological phenomena, is its alignment with those phenomena. If qualitative mathematics is ever to be utilized effectively, a primary issue must be addressed: How can we determine whether a certain mathematical structure is an appropriate representation of a phenomenon? There are significant debates about whether quantitative structures accurately represent psychological phenomena, and similar discussions could arise with other structures. A critical unresolved question is how to verify if ideas inspired by intuition are correct. Without an answer to this, the practical implementation of qualitative mathematics in psychology remains limited.

Qualitative (pure) mathematics is characterized by its attitude towards its subject matter. Therefore, applying qualitative mathematics in psychology involves searching for mathematical structures that match psychological phenomena. However, employing a specific mathematical structure in a manner that aligns with a psychological structure could be difficult, as we might lack a method to determine whether it truly fits. Another related issue is whether psychological phenomena should or even could be aligned with any mathematical structure at all. It’s possible that there is no way to convincingly align some mathematical structures with psychological attributes or phenomena.

Psychological concepts are often vague, leading to questions about their existence and their ability to be thoroughly mathematized. It might be useful to remember that mathematical methods are tools for developing models, not direct representations of reality (Eronen and Romeijn, 2020), because mathematical models cannot perfectly represent reality (Bouleau, 2013). It is quite likely that for a large portion of psychological phenomena, there will be no suitable mathematical models, for other part, there will be a model applicable at a specific point in time, but the phenomenon will not be consistent and will vary with changes in time, and for another portion, there may be some mathematical models, but these could only be used as approximations of reality. Therefore, discussions on how to formalize and mathematize phenomena, and how to prepare students for flexibility in their formalizations, should be coupled with the understanding that it is acceptable to abandon formalization when a phenomenon may not possess the necessary regularity to be formalizable.

## Data Availability

The original contributions presented in the study are included in the article/supplementary material, further inquiries can be directed to the corresponding author/s.
